# Postless versus traditional hip arthroscopy: A multilevel meta‐analysis of current evidence on efficacy and safety

**DOI:** 10.1002/ksa.70048

**Published:** 2025-08-28

**Authors:** Nikolai Ramadanov, Maximilian Voss, Jonathan Lettner, Robert Hable, Robert Prill, Roland Becker, Vanessa Twardy, Ingo J. Banke

**Affiliations:** ^1^ Center of Orthopaedics and Traumatology, Brandenburg Medical School University Hospital Brandenburg an der Havel Brandenburg an der Havel Germany; ^2^ Faculty of Health Science Brandenburg Brandenburg Medical School Theodor Fontane Brandenburg an der Havel Germany; ^3^ Faculty of Applied Computer Science Deggendorf Institute of Technology Deggendorf Germany; ^4^ Clinic of Orthopaedics and Sports Orthopaedics, School of Medicine and Health, TUM University Hospital Technical University of Munich Munich Germany

**Keywords:** hip arthroscopy, hip surgery, perineal post, traction table

## Abstract

**Purpose:**

To date, no meta‐analysis has systematically compared postless and post‐assisted hip arthroscopy (HAS). This underscores the need for a structured synthesis of current evidence. To address this gap, a multilevel meta‐analysis was conducted to systematically compare outcomes and complication rates of HAS performed with and without a perineal post.

**Methods:**

A comprehensive search of PubMed, Embase, Epistemonikos, and CENTRAL was completed on 20 July 2025. A frequentist multilevel meta‐analysis with random‐effects modelling and Hartung–Knapp adjustment was conducted. Outcomes were summarised as pooled mean differences and proportions with 95% confidence intervals.

**Results:**

Eight primary studies including 1880 hips were analysed. The pooled nerve injury rate was higher in the traditional HAS group (7%; 95% confidence interval [CI]: 0.01–0.36) compared to the postless group (3%; 95% CI: 0.00–0.20), with a significant subgroup difference (*F*  =  10.81; *p* < 0.01). Mean traction time was longer in the traditional group (58.5 min) than in the postless group (52.2 min), also with a significant difference (*F* =  32.96; *df* = 1.50; *p* < 0.01). Other subgroup comparisons showed no significant differences.

**Conclusion:**

While trends suggest potential advantages of postless hip arthroscopy in certain outcomes, the evidence remains limited by study heterogeneity and design. These results support its growing clinical use, though further prospective comparative studies are needed to strengthen the evidence base.

**Level of Evidence:**

Level II, systematic review and meta‐analysis.

**Abbreviations**
CENTRAL: Cochrane Library's Central Register of Controlled Trials
CI: confidence interval
FAIS: femoroacetabular impingement syndrome
GRADE: Grading of Recommendations, Assessment, Development, and Evaluation
HAS: hip arthroscopy
MCID: minimal clinically important difference
PRISMA: Preferred Reporting Items for Systematic Reviews and Meta‐Analyses
PROM: patient‐reported outcome measure
PROSPERO: International Prospective Register of Systematic Reviews
ROBINS‐I: Risk of Bias in Non‐randomised Studies of Interventions
VAS: visual analogue scale

## INTRODUCTION

Hip arthroscopy (HAS) has become a widely adopted technique for the treatment of femoroacetabular impingement syndrome (FAIS) and related intra‐articular pathologies. Traditionally, distraction of the hip joint during HAS is achieved using a perineal post, which provides countertraction and enables access to the central compartment. However, the use of a perineal post has been associated with a range of complications, including pudendal nerve injury, perineal soft tissue damage, and genitourinary trauma, raising important concerns about its safety profile [21].

Recent literature suggests that the incidence of post‐related complications may be underestimated in retrospective reports. A systematic review demonstrated that prospective studies report a significantly higher rate of such complications compared to retrospective studies (7.1% vs. 1.4%), with some cases of permanent nerve injury [21]. These findings have stimulated interest in postless techniques, which aim to achieve effective distraction while minimising the risk of perineal harm.

Postless HAS utilises friction‐based stabilisation methods in combination with Trendelenburg positioning to create countertraction without the need for a perineal post [22]. The underlying biomechanical principles have been well‐described using free‐body diagrams and friction models, allowing for patient‐specific optimisation of traction force, bed angle, and safety measures [22]. Studies have demonstrated that sufficient distraction can be achieved with Trendelenburg angles as low as 3°–5°, and that the method is broadly reproducible across a range of body types and operating room setups [22].

A variety of postless techniques have been described in the literature, including the use of specialised pads, yoga mat configurations, and even standard operating room equipment without the need for additional hardware purchases [3]. Clinical results from these techniques have been encouraging, with extremely low rates of pudendal neurapraxia and no reports of major complications [3]. Moreover, postless approaches are now being routinely employed in several institutions, reflecting a broader shift in surgical practice.

This trend is further supported by recent international surveys among hip preservation specialists, which demonstrate a growing adoption of postless hip arthroscopy—primarily motivated by the desire to reduce pudendal nerve and soft tissue complications. Surgeons who transitioned to postless techniques reported significantly fewer traction‐related injuries and perceived patient recovery as equal to or better than with traditional post‐assisted setups. Collectively, these findings support the desired safety, reproducibility and clinical feasibility of postless hip arthroscopy across a wide range of practice environments [10, 23].

Postless hip arthroscopy may involve higher costs due to the use of single‐use materials or specialised padding systems. However, if the technique proves to be safer—and potentially more effective—its adoption may be well justified. In particular, reduced complication rates and improved patient comfort could offset the initial material expenses in the long term.

Despite the increasing adoption of postless HAS, the comparative safety and efficacy of post‐assisted versus postless techniques remain incompletely understood. To date, no meta‐analysis has systematically compared postless and post‐assisted HAS. This underscores the need for a structured synthesis of current evidence. To address this gap, a multilevel meta‐analysis was conducted to systematically compare outcomes and complication rates of HAS performed with and without a perineal post.

## METHODS

### Protocol registration and reporting standards

The study was registered in the International Prospective Register of Systematic Reviews (PROSPERO) on 7 June 2025 (CRD420251068473). The methodology followed the updated PRISMA (Preferred Reporting Items for Systematic Reviews and Meta‐Analyses) 2020 guidelines [15], and a completed PRISMA checklist is included as supplementary material (Supporting Information: Table 1).

### Search strategy

A comprehensive search was conducted in four electronic databases: PubMed, Embase, Epistemonikos, and the Cochrane Central Register of Controlled Trials (CENTRAL), with the final search performed on 20 July 2025. A Boolean strategy was applied using the terms (((hip arthroscopy) OR (arthroscopy) OR (HAS)) AND ((postless) OR (perineal post))), and the syntax was adjusted for each database accordingly.

### Study screening and eligibility

Two independent reviewers (N.R. and M.V.) screened the records in two stages: first by title and abstract, followed by full‐text assessment. Any disagreements were resolved through discussion until consensus was reached. Cohen's kappa (*κ*) was calculated to assess inter‐reviewer agreement. Studies were eligible if they were prospective or retrospective primary research comparing HAS performed with versus without a perineal post. Editorials, reviews, animal studies, case reports, and grey literature were excluded. Only studies reporting relevant clinical outcomes were included. Postless techniques were defined as those employing Trendelenburg positioning, friction‐based pads, or other standard operating room adaptations that did not involve a perineal post. Since a multilevel meta‐analytic model was used, studies based on the same patient cohort were not excluded; instead, only distinct outcomes were extracted from each study, and duplicate outcome data were excluded where applicable.

### Outcome measures

Primary outcomes comprised complication rates, including nerve injury and numbness and sexual and urinary dysfunction. Secondary outcomes included traction time and pain assessed by the visual analogue scale (VAS) [9].

### Data extraction and quality appraisal

Two reviewers (N.R. and J.L.) independently extracted data using a pre‐specified form. Extracted data included study design, sample size, patient characteristics, clinical outcomes, and follow‐up duration. The full data extraction sheet is provided as supplementary material (Supporting Information: Table S2). Risk of bias was assessed independently by both reviewers using the ROBINS‐I tool [19] for non‐randomised studies. Discrepancies were resolved through discussion. The overall certainty of evidence for each outcome was assessed using the GRADE framework [6].

### Statistical analysis and meta‐analytic model

A frequentist multilevel meta‐analysis was performed to estimate treatment effects comparing HAS with versus without a perineal post. A random‐effects model was used, applying inverse‐variance weighting and restricted maximum likelihood estimation, with Hartung–Knapp adjustment [17]. This multilevel model structure was used to account for dependencies arising from multiple outcomes reported within the same study or patient cohort. Continuous outcomes were summarised as pooled mean differences with 95% confidence intervals. Subgroup comparisons were performed to evaluate differences between techniques. Statistical heterogeneity was quantified using the *I*² statistic, categorised as low (<25%), moderate (25%–75%), or high (>75%). All analyses were performed using R, with the meta and metafor packages. A *p*‐Value < 0.05 was considered statistically significant.

## RESULTS

### Search results

**Figure 1:**
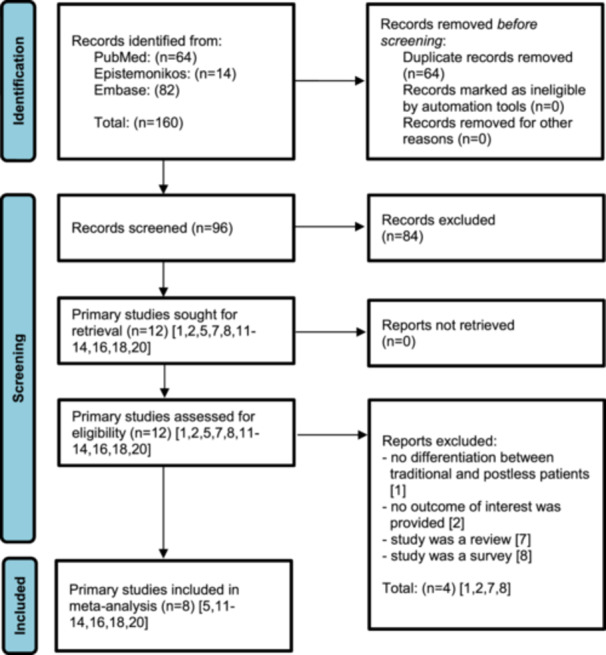
PRISMA chart flow diagram. Study selection process based on PRISMA 2020, including search results as of 20 July 2025. Eight studies [5, 11, 12, 13, 14, 16, 18, 20] were included in the final analysis. PRISMA, Preferred Reporting Items for Systematic Reviews and Meta‐Analyses.

A systematic literature search identified 12 studies [1, 2, 5, 7, 8, 11, 12, 13, 14, 16, 18, 20], all of which underwent full‐text screening (*κ* = 0.92). Of these, four studies [1, 2, 7, 8] were excluded for the following reasons (*κ* = 1.00): (1) inability to differentiate between post and postless patients [1], (2) lack of relevant outcomes [2], (3) review article [7] and (4) survey‐based study [8]. As a result, eight primary studies [5, 11, 12, 13, 14, 16, 18, 20] were included in the multilevel meta‐analysis (Figure 1). These studies encompassed a total of 1880 operated hips. Among them, five studies [5, 11, 12, 16, 18] reported on traditional HAS with a perineal post (*n* = 413 hips), while seven studies [11, 12, 13, 14, 16, 18, 20] focused on postless HAS (*n* = 1467 hips). The mean patient age across studies was 31.1 years (range: 26.1–38.1 years), with 47.3% male patients. The mean body mass index (BMI) was 25.2 kg/m² (range: 24.0–26.6 kg/m²) (Table 1).

**Table 1:** Study and patient characteristics.

Author	Journal	Study design	LoE	Indication	Patients, *N*	Hips, *N*	Age, years, SD (range)	Male sex, *N* (%)	BMI, kg/m², SD (range)	Follow‐up time, months
Güven et al. [5]	Orthopaedics and Traumatology: Surgery and Research	Retrospective cohort study	4	Traditional	118	118	38 ± 8 (18–50)	64 (54)	26 ± 4 (18–35)	3–12
Kraeutler et al. [11]	The American Journal of Sports Medicine	Prospective cohort study	2	Traditional	53	53	32 ± 14 (15–68)	24 (45)	25 ± 8 (16–45)	6
				Postless	34	34	27 ± 11 (14–51)	15 (44)	24 ± 9 (17–45)	
Kraeutler et al. [12]	The American Journal of Sports Medicine	Prospective cohort study	3	Traditional	41	41	34 ± 13	16 (39)	26 ± 7	24 ± 9
				Postless	28	28	29 ± 11	13 (46)	24 ± 4	21 ± 9
Mei‐Dan et al. [13]	Orthopaedics	Prospective case series	4	Postless	170	171	34 ± 65 (16–66)	111 (65)	25	6–12
Mei‐Dan et al. [14]	The American Journal of Sports Medicine	Prospective case series	4	Postless	NR	1000	NR	NR	NR	12–24
Parkes et al. [16]	The American Journal of Sports Medicine	Retrospective cohort study	3	Traditional	101	101	32 ± 8	45 (45)	24 ± 4	12
				Postless	94	94	30 ± 8	38 (40)	25 ± 5	
Schaver et al. [18]	The Journal of Arthroscopic and Related Surgery	Retrospective cohort study	3	Traditional	100	100	26 ± 9 (24–28)	42 (42)	27 ± 5 (26‐28)	<1
				Postless	100	100	27 ± 10 (25–29)	32 (32)	26 ± 6 (25–27)	<1
Welton et al. [20]	The American Journal of Sports Medicine	Prospective case series	4	Postless	35	40	32 ± 10 (16–53)	14 (40)		<1

Abbreviations: BMI, body mass index; LoE, level of evidence; NR, not reported; SD, standard deviation.

### Study characteristics

Güven et al. [5] conducted a retrospective cohort study of 118 patients undergoing HAS with a perineal post. The authors reported 14 cases of nerve injury and one case of urinary dysfunction. The average operating time was 145.9 min and the average traction time was 74.6 min.

Kraeutler et al. [11] analysed 87 patients in a prospective cohort study performed by the same surgeon at two institutions. The post group experienced significantly more cases of nerve injury (29 vs. 11) and sexual or urinary dysfunction (5 vs. 1) than the postless group. The average traction time did not significantly differ between the groups (post: 63 ± 20 minutes; postless: 55 ± 19 min). Kraeutler et al. [12] followed up the same cohort at 1 year and reported better outcomes in the postless group, for VAS 3.1 ± 2.7 versus 1.4 ± 1.6, and for mHHS 73.7 ± 16.7 versus 82.2 ± 12.2. Although Kraeutler et al. [11, 12] report on the same patient cohort, only unique outcome domains were extracted from each. Nerve injury data were retained only from the 2023 study to avoid duplication.

Although Mei‐Dan et al. [13] used a lateralized, proximal post, it did not apply countertraction to the perineum and was therefore classified as functionally postless. This classification was based on a detailed, well‐illustrated, and clearly described account of the surgical setup. No traction‐related complications were observed in this 170‐patient prospective case series. Mei‐Dan et al. [14] reported on 1000 hips treated postlessly. No groin‐related soft tissue or nerve complications occurred in any patient.

Parkes et al. [16] included 101 post and 94 postless patients in a retrospective cohort study. The post group had a significantly higher rate of nerve injury (45 vs. 29). The traction time was significantly lower in the postless group compared with the post group (43.89 ± 11.51 vs. 51.19 ± 10.06). Schaver et al. [18] compared 100 patients per group in a retrospective cohort study and reported no complications. The traction time and the operating time were significantly lower in the postless group compared with the post group (40.9 ± 11.1 vs. 45.8 ± 10.3; 89.1 ± 25.5 vs. 100.4 ± 17.9). Welton et al. [20] conducted a prospective case series in 35 postless patients, with no clinical perineal injury or urologic/sexual dysfunction observed.

### Quality assessment

The risk of bias across the included studies was evaluated using the ROBINS‐I tool (Table 2). Among the eight primary studies [5, 11, 12, 13, 14, 16, 18, 20] assessed, six [11, 12, 14, 16, 18, 20] were judged to have a moderate overall risk of bias, while two studies [5, 13] were rated as having a serious risk of bias, primarily due to concerns related to confounding and outcome measurement. Most studies showed a low risk in the domains of classification of interventions, deviations from intended interventions, and missing data. However, moderate bias was frequently observed in the measurement of outcomes, indicating limitations in blinding or reliance on subjective measures.

**Table 2:** Risk of bias assessment using the ROBINS‐I tool.

Study	Confounding	Selection	Classification	Deviations	Missing data	Outcome measurement	Reporting	Overall RoB
Güven et al. [5]	Serious	Moderate	Low	Low	Moderate	Serious	Moderate	Serious
Kraeutler et al. [11]	Moderate	Low	Low	Low	Low	Moderate	Low	Moderate
Kraeutler et al. [12]	Moderate	Low	Low	Low	Low	Moderate	Low	Moderate
Mei‐Dan et al. [13]	Serious	Moderate	Low	Low	Low	Moderate	Moderate	Serious
Mei‐Dan et al. [14]	Moderate	Low	Low	Low	Low	Moderate	Low	Moderate
Parkes et al. [16]	Moderate	Low	Low	Low	Low	Moderate	Low	Moderate
Schaver et al. [18]	Moderate	Low	Low	Low	Low	Moderate	Low	Moderate
Welton et al. [20]	Moderate	Low	Low	Low	Low	Moderate	Low	Moderate

*Note*: Possible judgement: low risk, moderate risk, serious risk, and critical risk. Most studies showed moderate overall risk of bias [11, 12, 14, 16, 18, 20] two studies [5, 13] were rated as having serious overall risk.

Abbreviation: ROBINS‐I, Risk of Bias in Non‐randomised Studies of Interventions.

### Publication bias

**Figure 2:**
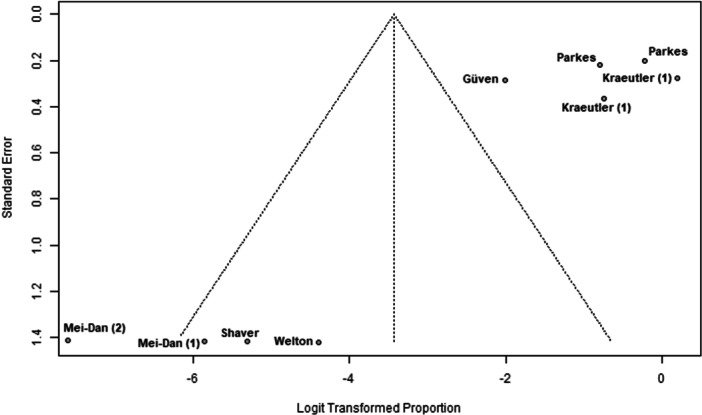
Funnel plot: Nerve injury and numbness. Each dot represents a study [5, 11, 13, 14, 16, 18, 20]*.* The asymmetry suggests potential publication bias.

**Figure 3:**
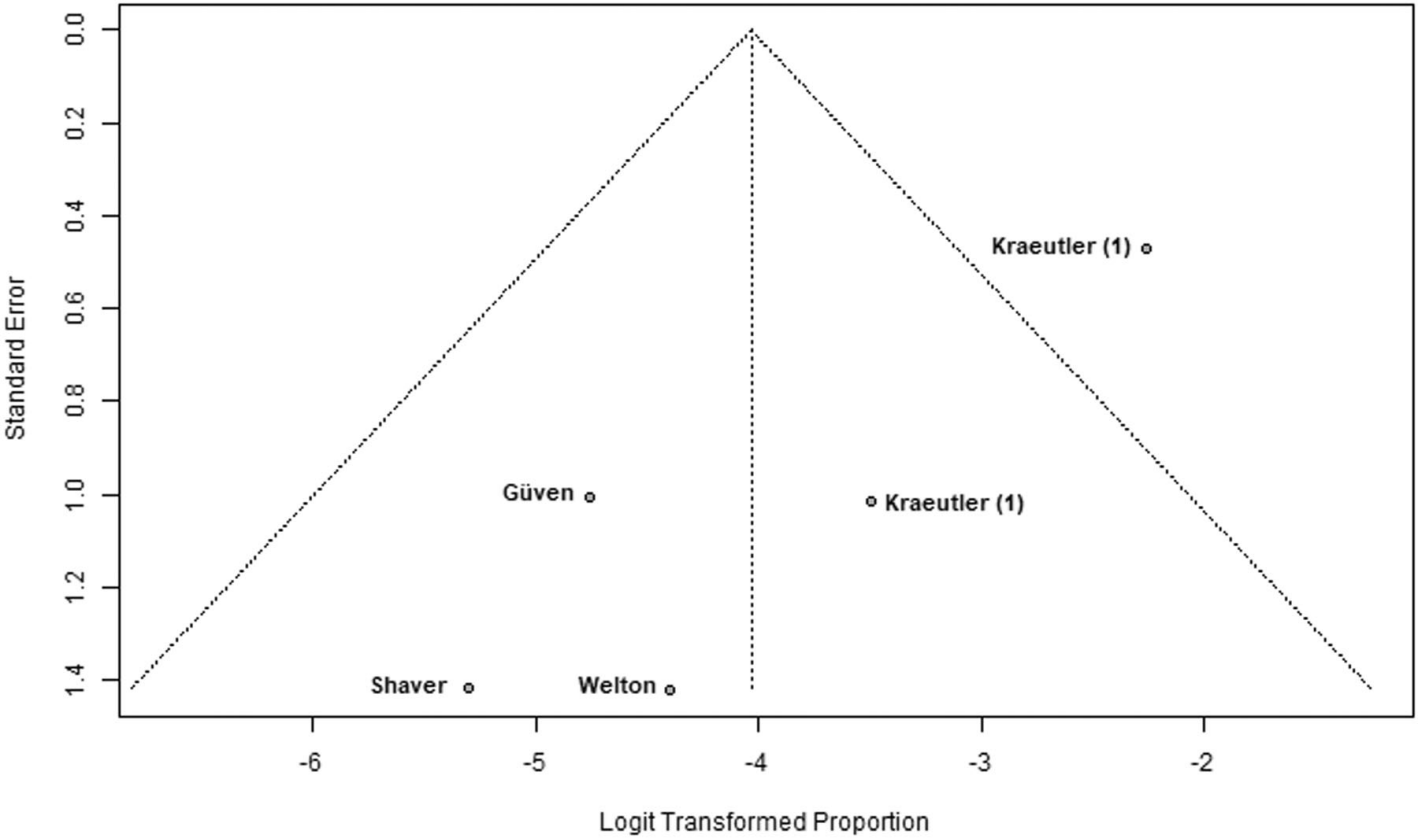
Funnel plot: Sexual and urinary dysfunction. Each dot represents a study [5, 11, 18, 20]*.* The asymmetry suggests potential publication bias.

**Figure 4:**
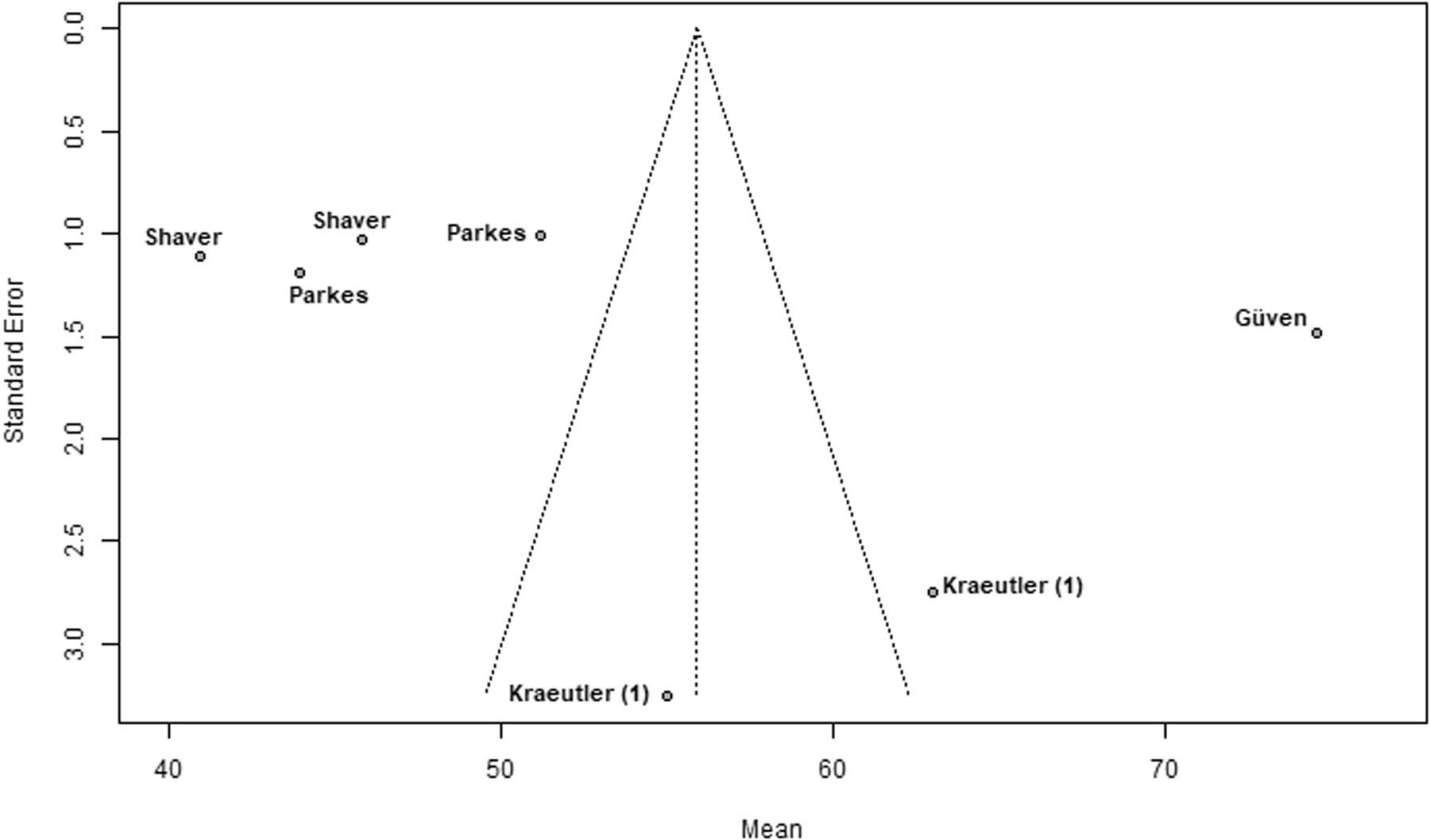
Funnel plot: Traction time. Each dot represents a study [5, 11, 16, 18]*.* The asymmetry suggests potential publication bias.

**Figure 5:**
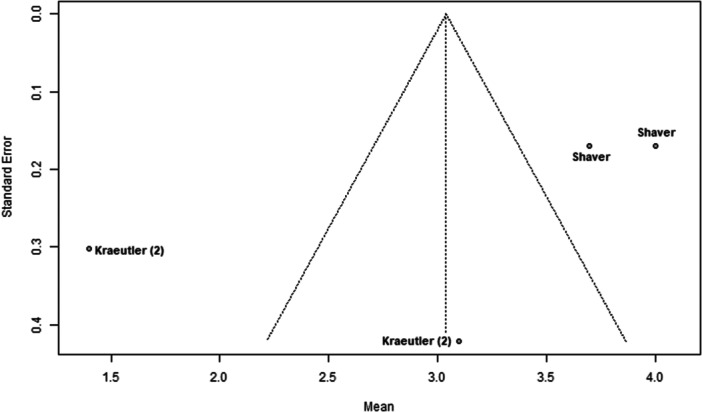
Funnel plot: VAS. Each dot represents a study [12, 18]*.* The distribution appears symmetric, suggesting no clear publication bias. VAS, visual analogue scale.

Funnel plots indicated potential publication bias in several outcomes. The plot for nerve injury and numbness showed clear asymmetry, suggesting reporting bias or heterogeneity (Figure 2). A similar pattern was observed for sexual and urinary dysfunction, with a lack of studies showing higher event rates (Figure 3). The plot for traction time also displayed dispersion, with studies like Güven et al. [5] reporting notably longer times (Figure 4). In contrast, the plot for VAS pain scores appeared more symmetric, indicating a lower risk of bias for this outcome (Figure 5).

### Multilevel meta‐analysis

#### Nerve injury and numbness

**Figure 6:**
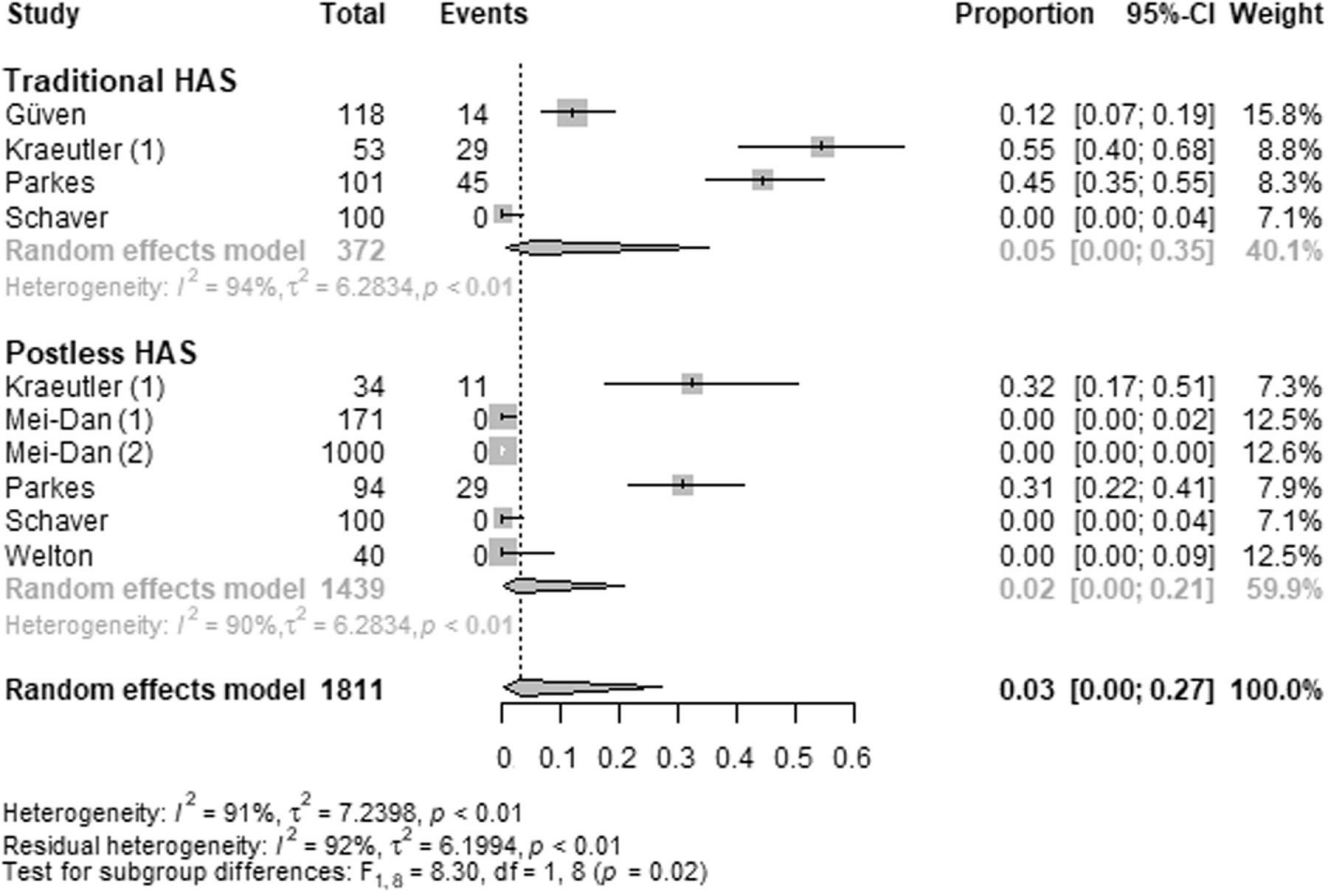
Forest plot: Nerve injury and numbness. Each line represents a study [5, 11, 13, 14, 16, 18, 20]. The traditional HAS group showed a significantly higher pooled proportion of nerve injury and numbness than the postless group. CI, confidence interval; HAS, hip arthroscopy.

The outcome parameter nerve injury and numbness was reported by seven primary studies [5, 11, 13, 14, 16, 18, 20], encompassing a total of 1811 operated hips (Figure 6 and Table 3). In the traditional HAS group, the pooled proportion of nerve injury and numbness was 5% (proportion = 0.05; 95% CI: 0.00–0.35; *I*² = 94%; *τ*² = 6.3; *p* < 0.01). In contrast, the postless HAS group showed a lower pooled proportion of 2% (proportion = 0.02; 95% CI: 0.00–0.21; *I*² = 89%; *τ*² = 5.6; *p* < 0.01). The test for subgroup differences indicated a statistically significant higher proportion of nerve injury and numbness in the traditional HAS group compared to the postless group (*F* = 8.30; *df* = 1.80; *p* = 0.02).

**Table 3:** Results of the multilevel meta‐analysis.

	Primary studies, *N*	Hips, *N*	Proportion	CI	*τ* ^2^	*I* ^2^	Heterogenity: *p*‐Value	Difference: *p*‐Value
Nerve injury and numbness								
Total	10	1811	0.03	0.00–0.27	7.24	0.91	<0.01***	0.02*
Traditional	4	372	0.05	0.00–0.35	6.28	0.94	<0.01***	
Postless	6	1439	0.02	0.00–0.21	6.28	0.90	<0.01***	
Sexual and urinary dysfunction								
Total	6	445	0.02	0.00–0.11	1.42	0.57	0.04*	0.34
Traditional	3	271	0.02	0.00–0.19	1.34	0.75	0.02*	
Postless	3	174	0.01	0.00–0.12	1.34	0.00	0.58	
Traction time								
Total	7	600	55.90	39.04–72.76	195.36	0.99	<0.01***	<0.01**
Traditional	4	372	58.45	42.14–74.76	158.58	0.99	<0.01***	
Postless	3	228	52.20	35.81–68.59	158.58	0.89	<0.01***	
VAS								
Total	4	269	3.04	0.41–5.67	1.61	0.95	<0.01***	0.58
Traditional	2	141	3.39	−0.75 to 7.52	1.75	0.43	0.19	
Postless	2	128	2.73	−1.36 to 6.81	1.75	0.98	<0.01***	

*Note*: The traditional HAS group showed a significantly higher rate of nerve injury and numbness and longer traction time (**). *Statistically significant, **highly significant, ***very highly significant difference.

Abbreviations: CI, confidence interval; VAS, visual analogue scale.

#### Sexual and urinary dysfunction

**Figure 7:**
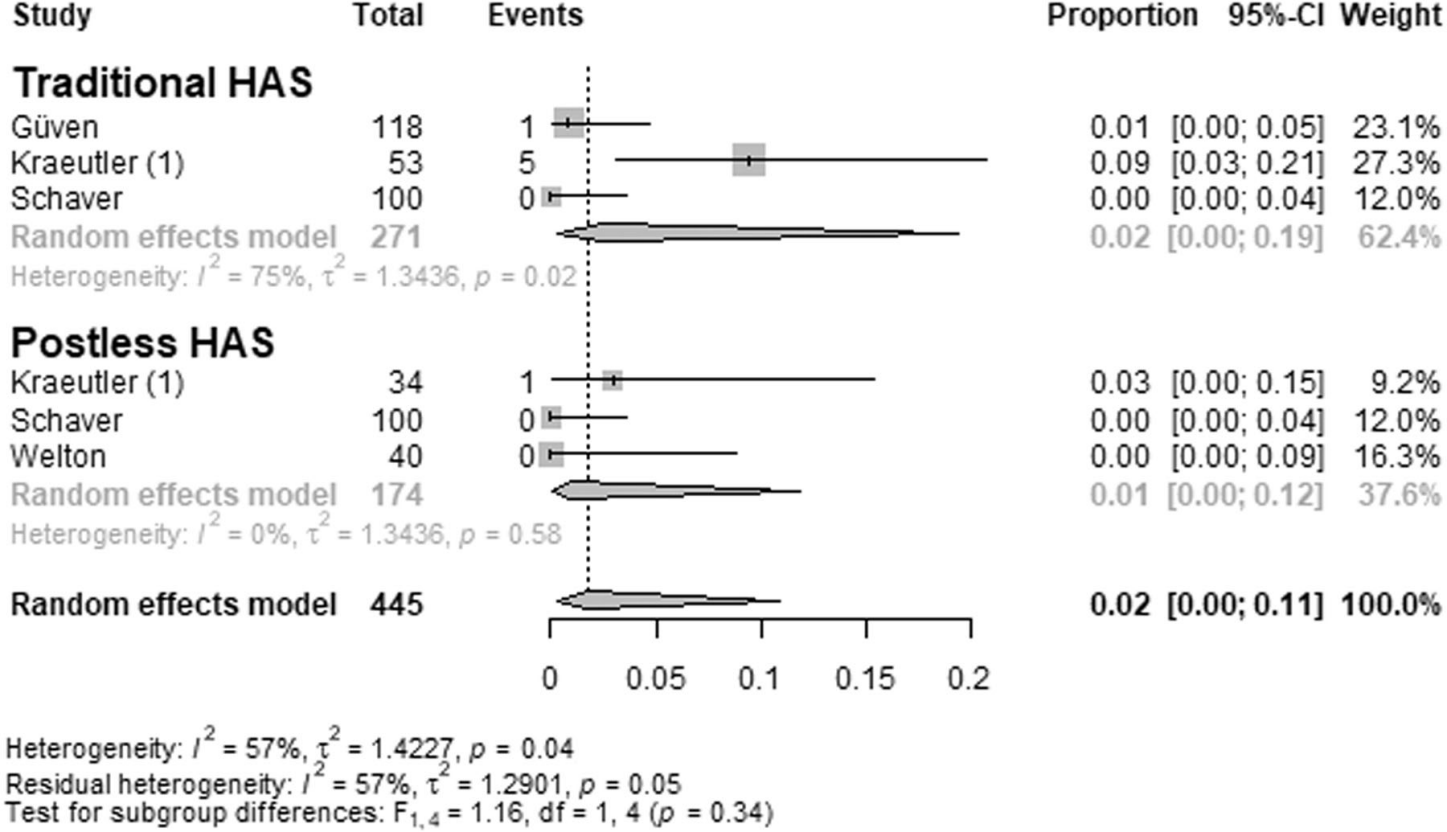
Forest plot: Sexual and urinary dysfunction. Each line represents a study [5, 11, 18, 20]. No significant difference in pooled proportions was found between groups. CI, confidence interval; HAS, hip arthroscopy.

The outcome parameter sexual and urinary dysfunction was reported by four primary studies [5, 11, 18, 20], encompassing a total of 445 operated hips (Figure 7 and Table 3). In the traditional HAS group, the pooled proportion of sexual and urinary dysfunction was 2% (proportion = 0.02; 95% CI: 0.00–0.19; *I*² = 75%; *τ*² =1.3; *p* = 0.02). In contrast, the postless HAS group showed a lower pooled proportion of 1% (proportion = 0.01; 95% CI: 0.00–0.12; *I*² =0%; *τ*² = 1.3; *p* = 0.58). The test for subgroup differences showed no statistically significant differences between the traditional HAS group compared to the postless group (*F* = 1.16; *df* = 1.40; *p* = 0.34).

#### Traction time

**Figure 8:**
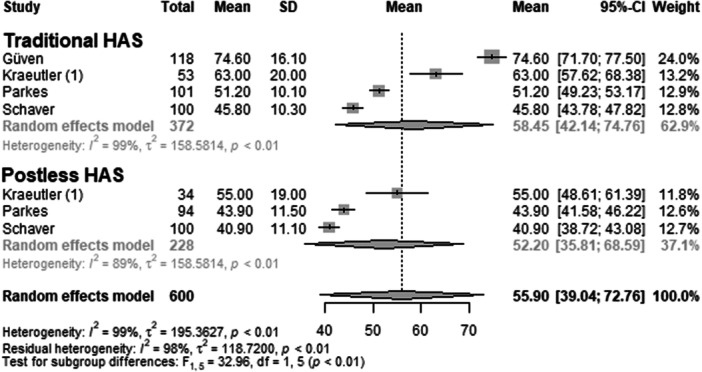
Forest plot: Traction time. Each line represents a study [5, 11, 16, 18]. The traditional HAS group showed significantly longer traction time compared to the postless group. CI, confidence interval; HAS, hip arthroscopy; SD, standard deviation.

The outcome parameter traction time was reported by four primary studies [5, 11, 16, 18], encompassing a total of 600 operated hips (Figure 8 and Table 3). In the traditional HAS group, the mean traction time was 58.5 min. (mean = 58.45; 95% CI: 42.14–74.76; *I*² = 99%; *τ*² = 158.58; *p* < 0.01). In contrast, the postless HAS group had a mean traction time of 52.2 min. (mean = 52.20; 95% CI: 35.81–68.59; *I*² = 89%; *τ*² = 158.58; *p* < 0.01). The test for subgroup differences indicated a statistically significant higher traction time in the traditional HAS group compared to the postless group (*F* =  32.96; *df* = 1.50; *p* < 0.01).

#### VAS

**Figure 9:**
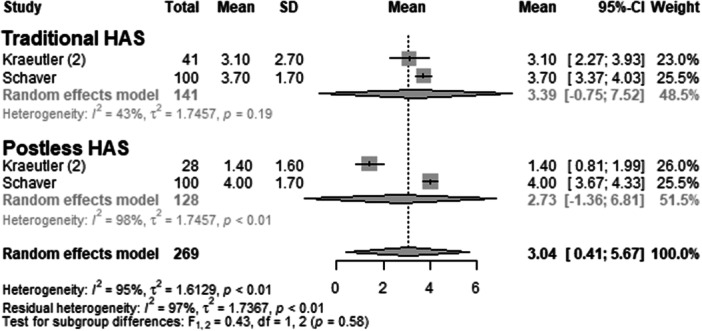
Forest plot: VAS. Each line represents a study [12, 18]. No significant difference in VAS scores was observed between groups. CI, confidence interval; HAS, hip arthroscopy; SD, standard deviation; VAS, visual analogue scale.

The outcome parameter VAS was reported by two primary studies [12, 18], encompassing a total of 269 operated hips (Figure 9 and Table 3). In the traditional HAS group, the mean VAS was 3.39 points (mean = 3.39; 95% CI: −0.75 to 7.52; *I*² = 43%; *τ*² = 1.75; *p* = 0.19). In contrast, the postless HAS group had a mean VAS of 2.7 points (mean = 2.73; 95% CI: −1.36 to 6.81; *I*² = 98%; *τ*² = 1.75; *p* < 0.01). The test for subgroup differences indicated no statistically significant differences in the traditional HAS group compared to the postless group (*F* = 0.43; *df* = 1.20; *p* = 0.58).

## DISCUSSION

This multilevel meta‐analysis provides a comprehensive comparison of HAS performed with versus without a perineal post, focusing on safety‐related complications and operative characteristics. Our findings indicate that postless HAS is associated with a significantly lower incidence of nerve injury and numbness compared to traditional post‐assisted techniques, without compromising operative efficacy. In addition, postless HAS was associated with significantly shorter mean traction times.

The risk of nerve injury and numbness was notably higher in the post‐assisted group (5%) than in the postless group (2%), a difference that was statistically significant [5, 11, 13, 14, 16, 18, 20]. These results align with prior reports suggesting that the perineal post may be a significant contributor to traction‐related neuropraxia, particularly involving the pudendal and peroneal nerves [21, 22]. The use of friction‐based stabilisation methods and Trendelenburg positioning in postless techniques may effectively eliminate this risk by avoiding direct perineal pressure.

Similarly, although rates of sexual and urinary dysfunction were low across all studies, the observed trend again favoured postless HAS, with half the proportion compared to post‐assisted techniques [5, 11, 18, 20]. While the subgroup difference was not statistically significant, the minimal heterogeneity observed in the postless group supports the consistency of this outcome and highlights the safety profile of postless configurations.

Postless HAS was associated with a significantly shorter traction time compared to procedures performed with a perineal post [5, 11, 16, 18]. This difference may reflect increased surgical efficiency when using postless positioning systems, potentially due to simplified patient setup or reduced need for intraoperative adjustments. However, traction time is highly dependent on surgeon experience, technique, and case complexity, which may introduce variability.

Pain outcomes, as measured by VAS scores, did not differ significantly between groups [12, 18]. Although the point estimate slightly favoured the postless group, the wide confidence intervals and heterogeneity—particularly in the postless group—limit firm conclusions. Importantly, the absence of a difference suggests that omitting the perineal post does not negatively affect early postoperative pain control.

In terms of operating time, postless HAS was associated with significantly shorter mean surgical durations [12, 18]. This could reflect a more streamlined setup, reduced need for repositioning or intraoperative adjustments, improved fluoroscopic visualisation, or greater surgeon familiarity with the technique in more recent studies. Although the absolute time savings were modest, the difference was statistically significant and may have implications for operating room efficiency.

These findings are consistent with recent survey‐based investigations into global surgical practice patterns. Volpi et al. [23] reported significantly lower rates of complications—including nerve injuries and genital skin trauma—among surgeons using postless distraction, with 97% stating that recovery was equal or improved compared to post‐assisted techniques. Similarly, Kraeutler et al. [10] found that among hip arthroscopists who had transitioned to postless techniques, the majority observed a decrease in pudendal and soft tissue complications. These large international surveys not only corroborate the lower complication rates associated with postless traction observed in our meta‐analysis but also underscore a growing clinical shift toward postless configurations in hip arthroscopy. Previous studies, such as Dippmann et al. [4], have contributed important insights into traction‐related complications—particularly nerve dysfunction associated with perineal post use—even though traction setup was not their primary focus or a point of comparison.

### Clinical implications

The clinical adoption of postless HAS offers clear benefits in terms of reducing traction‐related complications, particularly nerve injuries and urogenital dysfunction. These advantages make it an appealing option, especially for younger and more active patients where minimising iatrogenic harm is paramount. However, despite its favourable safety profile, the widespread implementation of postless techniques may be limited by practical and economic considerations. Most postless systems rely on friction‐based stabilisation, which typically requires specialised positioning equipment, Trendelenburg‐compatible surgical tables, or commercial traction pads—resources that may not be available in all settings. Additionally, these setups can be costlier than traditional traction tables with perineal posts. Therefore, while postless HAS represents a promising step forward in procedural safety, its routine use must be balanced against institutional resources, case volume, and cost‐effectiveness considerations.

### Limitations and strengths

This review has several limitations and strengths: (1) The majority of included studies were observational, introducing risks of selection bias and residual confounding. (2) One key limitation of this meta‐analysis is the inclusion of both prospective [11, 12, 13, 14, 20] and retrospective studies [5, 16, 18], which introduces clinical and methodological heterogeneity. While this limits the strength of pooled conclusions, it reflects the current state of the literature, where high‐level comparative data are scarce. As such, our findings should be interpreted as exploratory and hypothesis‐generating. (3) Variation in how outcomes such as nerve injury or sexual dysfunction were defined and measured limits comparability. (4) Heterogeneity was high for several outcomes, particularly traction time and VAS, which may reflect differences in surgeon experience, technique, or patient populations. (5) Some included studies originated from the same authorship group and may be based on overlapping patient populations. In particular, Kraeutler et al. [11, 12] report on the same cohort but address different outcome domains; duplicate outcomes (nerve injury) were excluded to avoid bias. The studies by Mei‐Dan et al. [13, 14] used distinct distraction setups and operating protocols, suggesting that different cohorts were analysed. Nevertheless, residual overlap cannot be fully excluded and should be considered when interpreting pooled estimates. (6) One large postless cohort study [14] accounts for a substantial proportion of the total patient population. While this study is methodologically sound and transparently reported, its size may disproportionately influence the pooled effect estimates. (7) Despite efforts to define postless techniques consistently, some variation in implementation likely exists. (8) We employed a multilevel meta‐analytic model, which accounts for non‐independence between multiple outcomes reported within the same study or patient cohort. This framework allows us to include different outcome domains from the same source without violating statistical assumptions. (9) Our search was limited to studies explicitly addressing the traction method (perineal post, postless, or both) in the title, abstract, or keywords. This was done to ensure comparability between techniques. As a result, relevant studies that report post‐related complications without highlighting the traction method—such as Dippmann et al. [4]—were not captured. Including all such studies would have disproportionately increased post‐related data without adding meaningful postless comparisons, thereby biasing the review focus.

## CONCLUSION

While trends suggest potential advantages of postless hip arthroscopy in certain outcomes, the evidence remains limited by study heterogeneity and design. These results support its growing clinical use, though further prospective comparative studies are needed to strengthen the evidence base.

## AUTHOR CONTRIBUTIONS

Nikolai Ramadanov, Maximilian Voss and Jonathan Lettner performed the literature search, the data extraction and the risk of bias assessment. Robert Hable and Nikolai Ramadanov conducted the statistical calculations. Nikolai Ramadanov and Robert Hable created all figures and tables. Nikolai Ramadanov wrote the manuscript. Nikolai Ramadanov and Vanessa Twardy revised the manuscript. Ingo J. Banke, Robert Prill and Roland Becker supervised the work.

## CONFLICT OF INTEREST STATEMENT

The authors declare no conflicts of interest.

## ETHICS STATEMENT

Clinical trial no: CRD420251068473.

## Supporting information

Supplementary Table 1: PRISMA checklist.

Supplementary Table 2: Data extraction sheet.

## Data Availability

The raw data extraction set is provided in Supporting Information Appendix.
